# GPS sensor-based mobile learning for English: an exploratory study on self-efficacy, self-regulation and student achievement

**DOI:** 10.1186/s41039-015-0024-y

**Published:** 2015-11-17

**Authors:** Jerry Chih-Yuan Sun, Kai-Yu Chang, Yang-Hsueh Chen

**Affiliations:** 1 Institute of Education, National Chiao Tung University, 1001 Ta-Hsueh Road, Hsinchu, Taiwan; 2 Department of Education, National University of Tainan, 33, Sec. 2, Shu-Lin Street, Tainan, Taiwan

**Keywords:** Context-aware learning, Ubiquitous learning, Technology-enhanced language learning

## Abstract

This study investigated the effects of self-efficacy and self-regulation on student achievement in a context-aware learning environment. Particularly, an innovative global positioning system (GPS) sensor-based mobile learning system was used to facilitate English learning of different plants on campus. A total of 41 university students participated in the study, divided into high and low groups of self-efficacy and self-regulation. The findings showed that both self-regulation and self-efficacy were significant predictors of learning achievement in the mobile context-aware learning (MCL) context. Moreover, while the GPS-based MCL learning session had positive effects on learning achievement, no significant increase in self-regulation or self-efficacy was found in either the high or the low group, supposedly due to the short duration of the activity. The participants found the system easy to use and useful, but they also raised critical concerns that can inform future improvements. We hope this exploratory study can serve as a starting point from which more interactive, user-friendly GPS sensor-based learning systems will be generated and more areas of application will be further explored to foster self-regulated, self-motivated ubiquitous learning of mobile learners.

## Introduction

Mobile learning has been highlighted by the 2010, 2011, and 2012 Horizon Reports (Johnson et al. 2012; Johnson et al. 2010; Johnson et al. 2011) as one of the most promising trends in higher education. Rapid advancements in cloud computing and the permeation of mobile devices in recent years have further pushed mobile learning into the era of mobile context-aware learning (MCL). A number of MCL-based systems have been developed for learners, with the ambition of realizing anytime, anywhere, and most of all, seamless/ubiquitous learning (Hwang and Wu 2014; Hwang et al. 2009; Hwang et al. 2011).

Self-efficacy and self-regulation are both crucial factors across different learning contexts (Sun and Rueda 2012). For self-regulation, research has shown that students’ learning achievement improved significantly after tutors facilitated self-regulation strategies (Montague 2007; Porath and Bateman 2006). Research has also shown that, with proper design, learners’ self-regulation could be enhanced with the assistance of technology, such as handheld devices and radio-frequency identification (RFID) (El-Bishouty et al. 2010; Puzziferro 2008; Sha et al. 2012).

While self-efficacy has been much studied in mobile learning and MCL research, learners’ self-regulation has not received equivalent research attention, as MCL studies that investigate self-regulation (e.g., El-Bishouty et al. (2010)) are relatively few in number. In addition, most of the existing MCL research has utilized RFID (Hsu et al. 2011; Tan et al. 2007) technology; however, the potential of the global positioning system (GPS) could be investigated further. Compared to RFID, GPS’s wide sensing range may help learners engage in learning activities without the problems of finding RFID tags. When Abowd et al. (1997) conducted their MCL study with GPS, they concluded that the wireless communication still needed improvement so that it can be seamlessly integrated with the MCL. With the recent development, GPS is now readily applicable to most smartphones without the need to purchase and allocate RFID devices. Based on the above rationale, the purposes of this study are threefold: firstly, to examine the effect of self-efficacy and self-regulation on learning achievement in a GPS-based MCL; secondly, to explore the overall effectiveness of the GPS-based context aware mobile system on students’ learning achievement for English; and thirdly, we are interested in understanding participants’ user experience and attitudes toward the system for future improvement of the system.

## Literature review

### MCL

Context-aware learning, by definition, refers to the situation in which learners enter real-life situations so that they can directly immerse themselves in the environment and sharpen their skills. Context-aware learning regards context as part of the learning content, and being in a real-life environment will facilitate the learning process (Schank and Kass 1996). The aim is to encourage learners to investigate knowledge and learn to use it in a realistic and appropriate environment (Brown et al. 1989; Herrington and Oliver 1995). Even though context-aware learning has created potential for authentic learning and performance, Brown et al. (1989) remind us that a guide is indispensable to helping learners understand the purpose and procedure, as well as the requisite strategies to resolve ill-defined problems. Through such guidance and support, learners are more able to exploit and reflect on their learning progress, as well as understanding how they can apply what they have learned to different real-life situations through the learning by doing approach.

In the past, context-aware learning was frequently linked to teaching outside of the classroom, such as holding exhibitions or visiting museums or ecological parks. With the advancements in mobile technologies and the prevalence of mobile devices such as smartphones, tablets, and devices such as Google Glass, the traditional context-aware learning has evolved into mobile context-aware learning (MCL). Via MCL, learners are further allowed to send and receive information, and interact with others anywhere using their mobile devices. More specifically, learners’ locations and ambient information (e.g., temperature, altitude, etc.) can be detected so that relevant information can be provided to them for immediate consultation and seamless learning. As stated by Chang et al. (2011), mobile context-aware systems have the advantages of allowing learners to experience and observe real-life situations and so acquire knowledge to a fuller extent. Learners are more able to grasp the concept of abstract contents, in turn bringing about improved learning and performance.

Regarding research in this area, Zhao et al. (2008) developed the Mobile Learning Assistant for Mathematics that was adaptive to users’ preferences, learner characteristics, and the contextual environment. Particularly, adaptive contents were provided based on device capabilities and learners’ experience, and users were able to post questions (text message, photos, and video/audio captured by the mobile device) to participate in group discussion. The results showed that the users’ learning efficiency, as well as their motivation, increased as a result of using such an adaptive MCL approach. Hwang et al. (2009) applied MCL to the laboratory context. In the past, difficulties in operating laboratory instruments (e.g., performing single-crystal X-ray diffraction) had led to a high demand for experienced professionals to be on site so as to guide inexperienced operators. With the innovative context-aware expert system, appropriate steps and actions were displayed logically and systematically on mobile screens to novice operators. This approach was proved to reduce operational/experimental errors and decrease labor costs, as well as increasing the students’ overall learning efficacy.

In Abowd et al. (1997) research, GPS was integrated with their mobile context-aware tour guide (Cyberguide), including four components: map, information, positioning, and communications. They concluded that GPS is particularly useful and effective for outdoor learning activities. However, at that time, the wireless communication and the bandwidth were limited, which affected the efficiency of the GPS positioning. With the recent advancements in mobile devices, context-aware technology has become an important feature for mobile-assisted seamless learning or ubiquitous learning (Wong and Looi 2011).

Another line of MCL research focuses on users’ perceptions of and attitudes toward the MCL systems. Studies have shown that learners’ perceived ease of use and perceived usefulness of a system have a significant impact on their intention to use and actual usage of MCL systems (Huang et al. 2012). Tan et al. (2007) developed a ubiquitous learning system to facilitate outdoor teaching for elementary school students. In addition to improved learning performance, significant changes were observed in terms of users’ perceived ease of use and usefulness. Hsu et al. (2011) used RFID to create a ubiquitous learning system for elementary school students to learn about plants. Similar to Hsu et al. (2011) results, participants’ learning performance increased significantly; meanwhile, the learners found the system both easy to use and useful. Chen and Huang (2012) also used RFID to develop a context-aware ubiquitous learning system (CAULS) for 80 grade 6 students to learn in a museum. Results showed that students using CAULS system had a higher achievement than those of the control group, and they thought that the CAULS system was easy to use and was useful in learning. Based on the above results, we think it is possible that perceived ease of use and perceived usefulness, the two factors of user perception, may have positive impacts on users’ learning and performance. These two factors are also useful for assessing the usability of the MCL systems. As such, we evaluated participants’ perceptions of these two factors in order to improve the self-developed GPS-based MCL system.

### Self-regulation

Self-regulation refers to the awareness and set of behaviors adopted and practiced by a learner during the learning process of achieving a desired goal (Schunk et al. 2013). Self-regulation is not a mental ability or a skill for good academic performance; rather, it is a method involving the use of different strategies or goal setting so that learners can enhance their learning performance (Zimmerman 2002). Self-efficacy is individualized because learners have different abilities, traits, and personal goals. According to Schunk et al. (2013), when learners use self-regulatory strategies, they are able to learn more effectively and achieve set goals more easily. It is self-regulation that helps learners maintain self-awareness and guides their behaviors to reach their goals.

Previous attempts to facilitate learner self-regulation have been largely confined to traditional classrooms and student counseling. Zimmerman (2008), however, argued that approaches to self-regulated learning should be renewed to adapt to technology-enhanced learning environments in the twenty-first century. Research approaches should also be updated with new paradigms. In practice, specific strategies should be explored and validated for different contexts and pedagogical methodologies, such as learning systems that incorporate MCL. One example is the mobile context-aware and adaptive learning schedule (mCALS) tool developed by Yau and Joy (2008). This system allows learners to denote their location, identify whether their learning progress is on schedule, and proactively process unlearned content. Another context-aware learning system developed by El-Bishouty et al. (2010) helps undergraduates identify the different components within a personal computer so that they can assemble one on their own. This system provides learning content in accordance with the context and allows learners to adjust the contents, rendering flexibility, learner control, and personalized self-regulation strategies.

In summary, learning systems based on mobile learning and MCL can enhance learners’ abilities to self-regulate and use the appropriate strategies, thus assisting them in the learning process.

### Self-efficacy

Self-efficacy originates from Bandura’s social cognitive theory (Bandura 1982), which states that external environments and the behaviors of others affect individuals’ mentality and behaviors. Through observation and social interaction, individuals formulate their self-judgment of their competence to perform a specific task. This self-judgment leads the individual to decide on the amount of effort to invest in completing a task or to abandon it even before attempting it (Bandura 1977, 1982).

The self-efficacy construct has been investigated in MCL research. Studies have found that self-efficacy affects individuals’ use of mobile devices to undertake MCL, as those with a relatively higher self-efficacy for mobile devices are more willing to make use of such devices to learn and vice versa (Kay and Knaack 2005; Tsai et al. 2010). A study by Kay and Knaack (2005) focused on trainee teachers who learned using laptops and wireless Internet access. They found that the long-term application of this learning method increased the trainee teachers’ self-efficacy in the use of laptops and their general computer ability, while it facilitated their future use of computers as teaching aids.

Self-efficacy was found to influence a variety of learning outcomes in MCL contexts. In Tsai et al.’s study (Tsai et al. 2010), elementary students in the third to sixth grades used MCL with plants in their school using a personal digital assistant (PDA) and undertook book-based research before completing a questionnaire on self-efficacy. Learners with higher self-efficacy and confidence in using the PDA were better at learning with it. Furthermore, when a person’s self-efficacy for mobile devices increased, their anxiety for using such devices reduced (Kwon et al. 2007; Tsai et al. 2010). Hwang et al. (2011) developed a PDA-based context-aware learning system for elementary school students to observe butterflies in a real garden. In addition to significant improvements in learning achievements, there was also an increase in self-efficacy for using computers to learn.

Chularut and DeBacker (2004) consider that an effective learning strategy or method (for example, the use of concept maps) helps students to learn English and improve their self-efficacy for English. Wong (2005) also found that students who used such strategies performed better, thus leading to improvements in their self-efficacy for English. Their self-efficacy levels in turn affected their future learning performance and strategy use. Bouffard-Bouchard et al. (1991) found that self-efficacy is an important factor influencing self-regulation during the English verbal learning tasks. It follows that self-efficacy and self-regulation (e.g., goal setting, task strategies, help seeking, etc.) may be intercorrelated constructs. It would be of value to include both constructs in MCL studies to explore their joint effects on learning outcomes.

### The research gaps

**Fig. 1:**
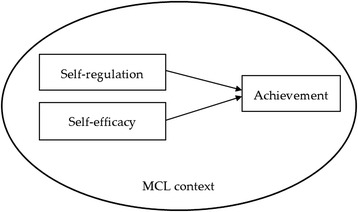
The research model

A review of the literature showed that, while self-efficacy has been much studied in mobile learning and MCL research, learners’ self-regulation has not received equivalent research attention. We contend that it would be a worthwhile endeavor to explore the role of self-efficacy and self-regulation together within the MCL learning context. Another research gap we found was that most of the existing MCL research has utilized RFID (Chen and Huang 2012; Hsu et al. 2011; Tan et al. 2007) technology; however, the potential of GPS can be further explored. For example, the Cyberguide developed by Abowd et al. (1997) still needs improvement in the integration of GPS and real-time communication. Therefore, the purpose of this study was to investigate the effects of self-efficacy and self-regulation on student achievement in a GPS sensor-based learning environment. The general research model is depicted in Fig. 1, and the four guiding research questions are listed below:

1. To what extent does self-regulation affect learning achievement in the GPS-based MCL environment?
2. To what extent does self-efficacy affect learning achievement in the GPS-based MCL environment?
3. To what extent do self-regulation, self-efficacy, and learning achievement change over the duration of the GPS-based MCL learning activity?
4. What are users’ perceptions of and attitudes toward the GPS-based MCL system?

## Methodology

### Participants

The participants of this study were 41 students from a national university located in Northern Taiwan, comprising 10 (24.4 %) undergraduate students and 31 (75.6 %) graduate students. The convenience sampling method was used to recruit the participants. The male to female ratio was 16 to 25 (39.1 to 60.9 %). The participants were aged between 19 and 27 years. The majority (28 or 63.4 %) were enrolled in the Humanities College, including Education, Communication Studies, and Applied Arts, followed by Science and Engineering (Computer Science, Electrophysics, and Communications Engineering) with 11 participants (26.8 %) and Management Science with 2 participants (4.9 %).

Approximately half of the participants (51.2 %) had been using smartphones for between 1 and 2 years. Only six (14.6 %) had less than 1-year experience, while two (4.9 %) had never used a smartphone (Table 1). In terms of mobile device usage, 85.4 % of participants had more than 1-year experience. Thus, we were not concerned about a lack of familiarity having a negative impact on the survey results of self-efficacy for mobile devices.

**Table 1:** Participant usage of smartphone

Duration	Number	Percent
Less than 1 year	6	14.6
1 to 2 years	21	51.2
2 to 3 years	7	17.1
3 to 4 years	1	2.4
4 to 5 years	3	7.3
5 years and above	1	2.4
Never	2	4.9
Total	41	100.0

### The GPS-based MCL system

**Fig. 2:**
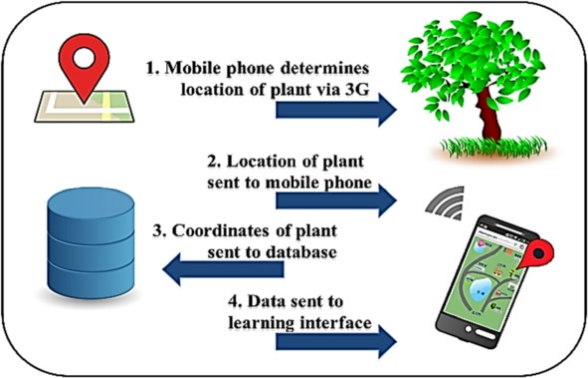
System configuration

**Fig. 3:**
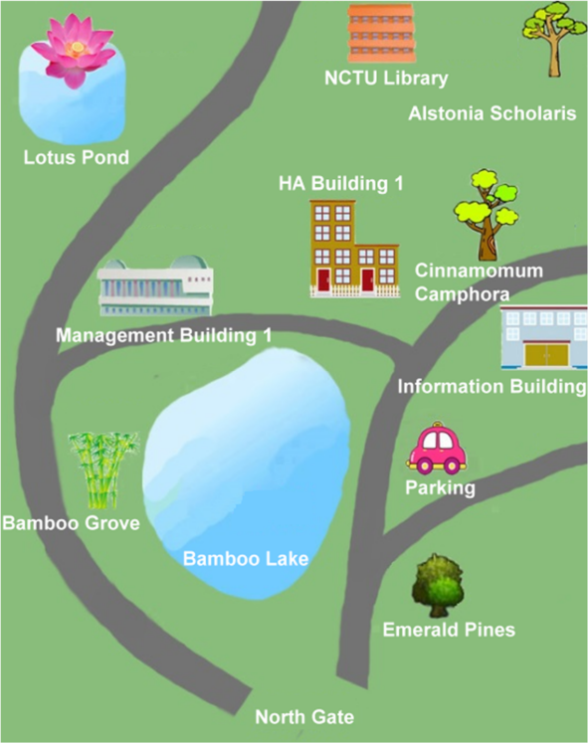
Mobile learning interface

**Fig. 4:**
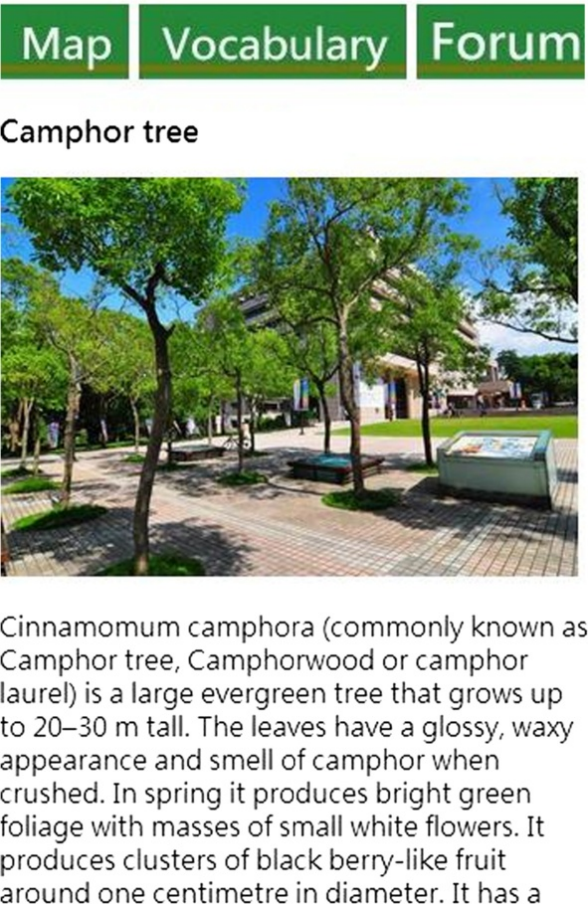
Mobile learning interface: contents

**Fig. 5:**
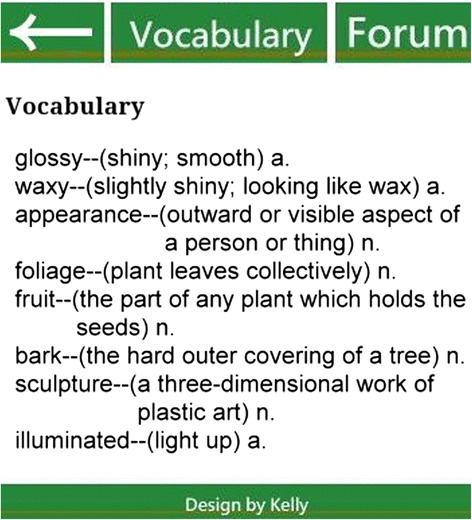
Mobile learning interface: words

**Fig. 6:**
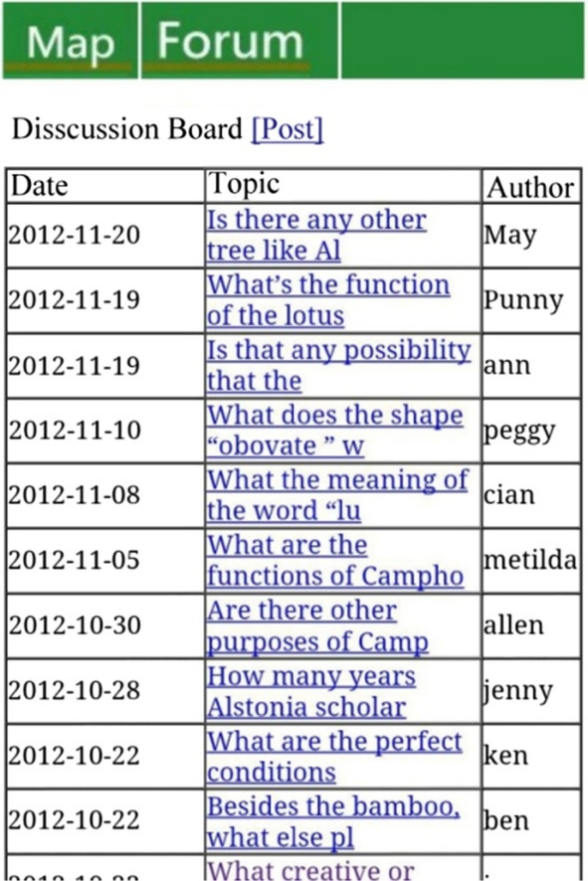
Mobile learning interface: forum

**Fig. 7:**
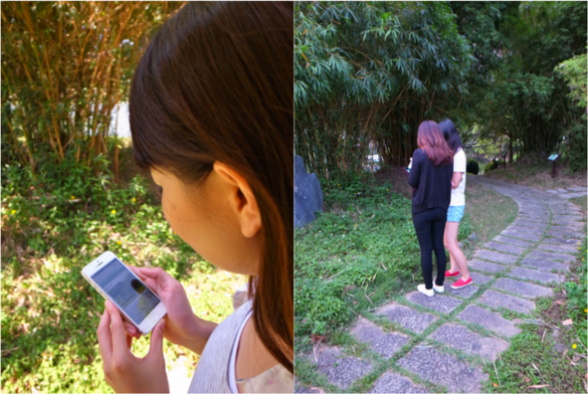
Photographs of learners undertaking mobile learning

In this study, we used a previously developed GPS-based MCL system for learning about plants on a campus (Sun and Chang 2014). As mentioned earlier, the strength of GPS technology is its wide sensing range. Also, compared to RFID or quick response (QR) codes, GPS users do not need to spend time finding QR codes or RFID tags. In addition, Looi et al. (2010) pointed out that the functions of the mobile devices are related to the type of learning activities. The feature of GPS is suitable for the wide learning environment such as the campus. With our GPS system, users’ positions are matched against previously determined learning locations stored in the system’s database. In the case of a match, the relevant learning content for the location will be automatically provided to the learners. Plants found on the campus were selected as the topic for the learning materials, adapted from the plants of Taiwan, an integrated query system on Taiwan’s botanical information. To facilitate interaction and collaborative learning, we further established a discussion forum wherein users could participate in real-time discussions when they were on site with their smartphones. The configuration of the system is shown in Fig. 2. Screenshots of the mobile learning interface, contents, and functions, as well as photographs of the real-life learning situation are shown in Figs. 3, 4, 5, 6, and 7.

### Instrumentation

The instruments used in this study include questionnaires on self-regulation, self-efficacy for English and mobile devices, perceived ease of use and usefulness of the system, and an achievement test on the plants. The Motivated Strategies for Learning Questionnaire (MSLQ) developed by Pintrich et al. (1991) was used to measure self-regulation, self-efficacy for English, and learning strategies. We translated the questionnaire according to the conditions of the study without revising the intended meaning of the questions. Prior to the formal study, experts helped examine the contents of the questionnaires translated into Chinese to ensure that the translated questions conveyed the intended meaning. The original MSLQ uses a seven-point scale. For consistency, we used a six-point Likert scale throughout this study, with 6 and 1 each denoting “strongly agree” and “strongly disagree.” The Cronbach’s alphas of self-regulation, self-efficacy for English, and learning strategies were .69, .88, and .83, respectively. The instruments used in this study were adapted from existing validated scales. We also measured participants’ self-efficacy for mobile devices. Items from Tsai’s (Tsai et al. 2010) PDA self-efficacy survey (PSS) were adapted and slightly modified to fit the purpose of this study. A sample question is “In the u-learning context, I think I can read the content on the screen using a smartphone.” The Cronbach’s alpha was .91. The confirmatory factor analysis (CFA) of model fit indicated χ^2^ = 6.50, *p* = .89, RMSEA = .00, CFI = 1, and SRMR = .03. Based on the model fit criterion proposed by Hu and Bentler (1999) (CFI > .95, RMSEA < .08, and SRMR < .08), a good fitting result and construct validity were obtained. The values of factor loadings were between .97 and .61, reaching the significant level of .05.

Participants’ perceived ease of use and perceived usefulness of the GPS-based MCL system were based on the Technology Acceptance Model (TAM) questionnaire proposed by Davis (1989). Items were slightly modified to fit the purpose of this study. Sample questions include “It would be easy for me to become skillful at using the GPS-based MCL System” (perceived ease of use) and “I would find the GPS-based MCL System useful in learning about different plants in English” (perceived usefulness). The Cronbach’s alphas were .89 and .86, respectively.

Lastly, participants’ learning achievement was measured by the self-developed plants in English test. The test contained ten items. A sample question is “In which month does the Blackboard Tree (Alstonia scholaris) bloom?” The difficulty level of the test on average was .74. The discrimination power of D value was .40 on average, indicating the quality of items was considered good (Ebel and Frisbie 1986).

### Procedures

**Fig. 8:**
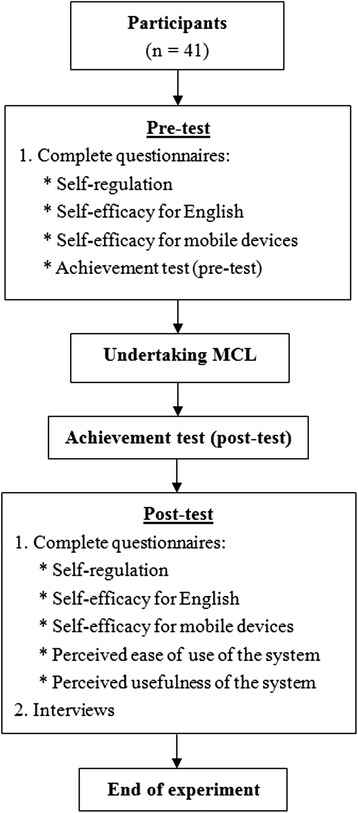
Logistics and flow of research

Figure 8 illustrates the logistics and flow of the study. Prior to using the MCL system, participants provided their demographic data and took pre-tests for self-regulation, self-efficacy (for English and mobile devices), and the achievement test. Then, we provided an orientation to the participants about the functions of the system, as well as how to complete the learning tasks. It is worth noting that we asked learners to observe (or even touch or smell) the designated campus plants, and search for related vocabulary about different parts of the plants or terms that describe the plants (e.g., glossy, waxy, etc.). We also encouraged participants to share what they observed or questions they had via the forum embedded in the system. A hand-sketched campus map was given to each participant as their learning aid.

The GPS-based context-aware mobile learning activity took about an hour. The participants went around the campus with their smartphones (see Figs. 3 and 7), matched their locations using the MCL system, and read the learning content and related guidance (see Fig. 4 as an example). After completing the session, they took the plants in English achievement test and completed post-test questionnaires on self-regulation, self-efficacy for English and mobile devices, and the perceived ease of use and usefulness of the GPS-based MCL system. Lastly, we individually interviewed all of the participants to obtain their feedback on the learning content and operation of the learning system.

The questionnaire data were analyzed using the SPSS statistics program. Statistical analyses included descriptive statistics, *t* test, analysis of covariance (ANCOVA), reliability tests, and regression analysis, which will be presented in more detail in the next section. Qualitative data collected from open-ended questions and interviews were used to understand the manner in which our GPS-based MCL system helped learners, the types of learning strategies used, and possible ways to improve the system and its contents for future use. Creswell’s (2008) six steps were referred to when analyzing the data:

1. Organize and prepare the data for analysis.
2. Read through all the data.
3. Begin detailed analysis with a coding process.
4. Use the coding process to generate a description of the setting or people as well as categories or themes for analysis.
5. Advance how the description and themes will be represented in the qualitative narrative.
6. Make an interpretation or meaning of the data.

## Results and discussion

### RQ1: To what extent does self-regulation affect learning achievement in the GPS-based MCL environment?

Table 2 presents the regression analysis results wherein learning achievement was regressed on self-regulation. We found that the learners’ self-regulation significantly predicted their post-test achievement scores ($\beta$ = 13.38, *p* < .01). That is, overall, learners with better self-regulation were more likely to achieve better plants in English. We further divided the participants into high (*N* = 20) and low (*N* = 21) groups based on their pre-test scores for self-regulation. As shown in Table 3, the analysis of covariance (ANCOVA, controlling for pre-test scores) results reveal that the high-self-regulation group performed better than the low-self-regulation group in terms of post-test achievement scores (*F*(1, 38) = 6.13, *p* < .05, *M*_HSR_ = 83.82 > *M*_LSR_ = 71.13).

**Table 2:** Regression analysis predicting learning performance from self-regulation

Predictors	Sum of square	*t*
Self-regulation	13.38 (0.44)	2.95**
Grouping for self-efficacy for mobile devices	3.70 (0.11)	0.74
Total R^2^		0.19
F value		4.37

**p < .01

**Table 3:** ANCOVA of post-test results for learners with high and low self-regulation

Source	Type I sum of squares	df	Mean square	*F*	*p*
Pre-test	265.03	1	265.03	1.09	.30
SRL post-test grouping	1492.74	1	1492.74	6.13	.02*
Deviation	9247.11	38	243.35		
Adjusted total	11004.88	40			

*p < .05

The above findings are in line with El-Bishouty et al. (2010) study wherein learners with better self-regulation performed better using the context-aware learning system. Our findings also echo Sha et al. (2012) study wherein more self-regulated students received higher achievement scores in their MCL learning context. As mentioned earlier, MCL studies that investigate learners’ self-regulation are relatively few in number. Our study adds evidence to the critical role of self-regulation in MCL learning. In the outdoor learning activity without instructor, self-regulation influenced learning performance. It is possible that the learners with high self-regulation employed the effective learning strategies and behaviors (e.g., posting questions on the discussion board, planning the route) during the limited learning time. More studies are suggested to examine detailed self-regulation strategies, such as goal setting, environment structuring, time management, help seeking, etc., to explore how these strategies could be facilitated to improve student achievement in the context of an MCL environment. At this point, we suggest that teachers or facilitators be attentive to learners’ self-regulation and provide differentiated learning tasks appropriate for them.

### RQ2: To what extent does self-efficacy affect learning achievement in the GPS-based MCL environment?

Table 4 illustrates the regression analysis results wherein learning achievement was regressed on self-efficacy. We found that the learners’ self-efficacy significantly predicted their post-test achievement scores ($\beta$ = 6.88, *p* < .05), showing that learners with better self-efficacy were more likely to achieve better plants in English. This finding is in accordance with previous studies such as Su and Duo (Su and Duo 2012). In their study, when learners improved their self-efficacy for English, their learning achievement increased as well. Chularut and DeBacker (2004) found that the use of well-devised teaching methods (e.g., concept mapping) helped improve English self-efficacy, in turn bringing about better learning performance. Chularut and DeBacker’s (2004) work reminds us that meticulously designed instructional strategies and learning aids could (or even should) be employed to leverage self-efficacy, self-regulation, and learning outcomes. Also, because most of the university students were not familiar with the learning content of plants in English, learners with higher levels of self-efficacy for English were more confident of understanding the learning materials and felt less anxious about learning in English and therefore had better performance. More studies are suggested in this direction, particularly in MCL learning environments.

**Table 4:** Regression analysis predicting learning performance from self-efficacy for English

Predictors	Sum of square	*t*
Self-efficacy for English	6.88 (0.35)	2.15*
Grouping for self-efficacy for mobile devices	4.89 (0.15)	0.91
Total *R* ^2^		0.11
F value		2.35

*p < .05

### RQ3: To what extent do self-regulation, self-efficacy, and learning achievement change over the duration of the GPS-based MCL learning activity?

Although we were cognizant of the fact that the learning activity only took 1 h, which may not be sufficient to bring about substantial changes to personal traits such as self-efficacy, still we were interested in exploring the extent to which the GPS-based MCL English might have influenced participants’ self-regulation, self-efficacy, and learning achievement. Table 5 provides some initial evidence of the overall effects of the GPS-based MCL learning activity on learning achievement. Although learners’ levels of self-efficacy for mobile devices did not have a significant impact on their learning performance, the achievement scores for both the high and low groups increased significantly (*p* < .001). This result indicates that our system was likely adapted to all learners, regardless of their level of self-efficacy for mobile devices, hence resulting in good performance overall.

**Table 5:** Comparison of pre- and post-test scores for learning performance within high and low self-efficacy for mobile groups

	High (*n* = 25)		Low (*n* = 16)	
	M (SD)	*t*	M (SD)	*t*
Pre-test	45.60 (13.25)	9.22***	50.63 (11.82)	4.57***
Post-test	76.80 (16.76)		78.13 (16.82)	

***p < .001

In terms of self-regulation, no significant difference between the pre- and post-tests was found (*p* = .47) (see Table 6). We suspect that, although we provided an orientation about the functions of the GPS-based MCL system, as well as how to complete the learning tasks such as observation, taking notes, or posting questions to the forum, the 1-h learning duration was still too short for students to fully apply self-regulated learning strategies, not to mention developing learning strategies of their own.

**Table 6:** Comparison of pre- and post-test results based on self-regulation

	M (SD)	*t*
Pre-test	4.29 (0.51)	0.76
Post-test	4.37 (0.54)	
Total N	41	

Not to our surprise, participants’ self-efficacy both for English and for mobile devices also yielded insignificant results (see Tables 7 and 8). Nevertheless, there was a slight improvement in the mean score of self-efficacy for English. This may be explained by the fact that the learning contents (plants in English) and the context (campus) were relevant to the learners’ lives. As such, they felt that they could easily grasp the contents and undertake learning successfully.

**Table 7:** Comparison of pre- and post-test results based on self-efficacy for English

	M (SD)	*t*
Pre-test	3.88 (0.81)	1.25
Post-test	4.03 (0.83)	
Total N	41

**Table 8:** Comparison of pre- and post-test results based on self-efficacy for mobile devices

	M (SD)	*t*
Pre-test	5.57 (0.66)	−0.12
Post-test	5.56 (0.62)	
Total *N*	41	

However, it was surprising to find that participants’ self-efficacy for mobile devices decreased from the pre-test to the post-test, although once again, the change was not statistically significant (see Table 8). It is possible that the participants had over-estimated their abilities prior to using the devices for learning. After using the GPS-based MCL system, they realized that their self-efficacy was not as high as they had previously thought. On the other hand, the result might also reflect some usability issues. The GPS MCL system might not be very user-friendly so that participants’ confidence decreased after using the system.

### RQ4: What are users’ perceptions of and attitudes toward the GPS-based MCL system?

The participants completed the perceived ease of use and perceived usefulness questionnaires after using the GPS-based MCL system. With the six-point scale adapted for the study, the mean scores of perceived usefulness and perceived ease of use were 4.79 and 5.17, respectively. Both scores were considered higher than the mean of 3.5 (Table 9), which indicated that, generally, the learners felt that the system was easy to use and useful in terms of learning about the plants in English.

**Table 9:** One-sample statistics

	Number	M	SD
Perceived usefulness	41	4.79	0.65
Perceived ease of use	41	5.17	0.56

We further conducted one-on-one interviews after the post-test to probe participants’ user experience, as well as their suggestions for improving the system. The majority of learners felt that there was excessive information on some pages, such as the Blackboard Tree (Alstonia scholaris) page and the Lotus Pond page. They found it overwhelming to read all of the information on their smartphone screens. Furthermore, although we strived to design the learning contents to be relevant to the participants’ daily life, some learners still felt that the contents could be livelier. Still others expressed that they wished to have done more than observing the plants (even after the orientation of learning strategies as mentioned earlier). They said the learning process could have been more dynamic by including various activities such as on-site interactions. Again, we think such feedback may be due to the short duration of the learning session which restricted their time to try out different ways of learning such as on-site interaction. Listed below are selected excerpts from the interviews:

‘Some parts of the content were excessive. The contents of the different sections could have been distributed more evenly. Try not to display too much information.’ (Participant 4)

‘Looking back on the entire experiment, I felt as though I was only looking at my mobile phone throughout the process, without any on-site interactions.’ (Participant 3)

Participants also commented on the forum embedded in our MCL system, with some saying that when they used it to post messages or questions on site during the learning session, they expected to receive instant responses from peers. However, they did not receive timely feedback to resolve their doubts, making them feel that the forum was ineffective. As such, in the future, we will revise our design of the learning tasks to facilitate on-site interactions, such as posting guiding questions on the forum beforehand. A few participants further suggested incorporating a search and query function so that they need not switch to another page. This comment provides valuable information for us to improve the user interface of our MCL system.

Unexpectedly, we realized that some learners were not very familiar with the campus environment. Although we gave the learners a simple hand-sketched map during the orientation, they still encountered difficulties finding the learning locations. They expressed that the map would have been more helpful if it indicated more buildings, locations, or landmarks. Two participants who had recently enrolled in the university even suggested providing an actual learning route.

Besides participants’ feedback, we also noted some problems or issues ourselves. Regarding the technical aspects of the GPS-based MCL system, it is ideal to set a wider range to avoid non-detection. Yet, the drawback is that it will take longer for the system to position and respond, especially when we are using different types of mobile devices or when many users are using the system. In the near future, we plan to figure out an exact balance point between detection and speed, meanwhile updating our server to increase the database processing capabilities for more users.

## Limitations and conclusion

This study has its limitations; foremost is that the duration of the learning session was only 1 h due to practical considerations. In the future, we will increase the time span so that the learners have sufficient time to go around the campus with their smartphones, match their locations using the MCL system, and apply more diversified learning strategies. Furthermore, this study is exploratory in nature, and we have not included a control group at this point. Future studies may employ an experimental design to closely examine the causal effects of the GPS-based MCL systems on learners’ self-regulation, self-efficacy, and learning achievement.

Despite the above limitations, this study utilizes a self-developed GPS mobile context-aware learning system to investigate the effects of self-regulation and self-efficacy on English learning. So, as to improve the system, we explored participants’ user experiences, including their perceived ease of use, perceived usefulness, and how it could be improved. Findings from this study may provide reference for the design and utilization of GPS sensor-based context-aware mobile devices in teaching English and related subjects. We hope this study can serve as a starting point from which more interactive, user-friendly GPS sensor-based learning systems will be generated and more areas of application will be further explored to foster self-regulated, self-motivated ubiquitous learning of mobile learners.
